# Paternal leakage of mitochondrial DNA is genetically controlled in Drosophila

**DOI:** 10.1093/g3journal/jkag199

**Published:** 2026-07-28

**Authors:** Maria-Eleni Parakatselaki, Stavroula Zapri, Sevasti Koursioti, Eirini Slavka Polovina, Emmanuel Ladoukakis

**Affiliations:** Department of Biology, University of Crete, Heraklion 70013, Crete, Greece; Department of Biology, University of Crete, Heraklion 70013, Crete, Greece; Department of Biology, University of Crete, Heraklion 70013, Crete, Greece; Department of Biology, University of Crete, Heraklion 70013, Crete, Greece; Department of Biology, University of Crete, Heraklion 70013, Crete, Greece

**Keywords:** mitochondrial DNA, paternal leakage, heteroplasmy, genome transmission, heritability, Drosophila, mitochondrial inheritance

## Abstract

Mitochondrial DNA is generally assumed to be strictly maternally inherited, with paternal leakage treated as a rare stochastic error. Using hybrids from multiple *Drosophila simulans* isofemale lines crossed to *D. mauritiana*, we assayed heteroplasmy and asked whether leakage has a genetic basis. Leakage varied consistently among lines and families, replicated across independent experiments, and was markedly higher in males than females. Estimates indicated high heritability, and natural isofemale lines showed greater among-family variance than inbred laboratory stocks. These results demonstrate that paternal mtDNA leakage is under strong genetic control, rather than resulting from stochastic errors in the mechanisms that enforce strict maternal transmission, suggesting potential functional implications for long-term mitochondrial genome stability and evolution.

## Introduction

Mitochondrial DNA (mtDNA) is traditionally regarded as strictly uniparentally inherited, a fundamental biological principle with only a few known exceptions in nature (Yang and Griffiths 1993; Apitz et al. 2013; Camus et al. 2022). In most organisms, mtDNA is transmitted maternally, although exclusive paternal inheritance has been documented in certain plant taxa, such as some Cucurbits (Havey 2017) and conifers (Adams 2019). The evolutionary pressure favoring uniparental transmission appears to be so strong that, in some bivalve species where both sexes can transmit mtDNA, 2 sex-specific, independently transmitted mitochondrial lineages coexist within the same species. This phenomenon is known as doubly uniparental inheritance (Zouros 2013).

The predominance of uniparental mtDNA inheritance remains a long-standing conundrum in evolutionary biology. One of the most widely accepted hypotheses proposes that uniparental transmission prevents genetic conflict between nonmeiotically propagated mtDNAs when more than 1 lineage coexists within the same cytoplasm. By ensuring genetic homogeneity, uniparental inheritance limits the spread of selfish mitochondrial genomes within populations (Cosmides and Tooby 1981; Hurst 1992; Birky 1995). Another hypothesis suggests that uniparental transmission enhances the coadaptation between mitochondrial and nuclear genomes (Hadjivasiliou et al. 2012).

The strong evolutionary pressure favoring uniparental mtDNA transmission is deduced by the diversity of the mechanisms that maintain this pattern (Sato and Sato 2017). These mechanisms include pre-fertilization destruction of mtDNA from spermatids during spermatogenesis or post-fertilization elimination of paternal mtDNA from the zygote, or both pre- and post-fertilization mechanisms. Particularly in *Drosophila melanogaster*, a pre-zygotic mechanism eliminates paternal mtDNA during spermatogenesis, involving the proteins EndoG (DeLuca and O'Farrell 2012) and POLDIP2 (Wang et al. 2025). Also, there is a post-fertilization mechanism which destroys paternal mitochondria immediately after fertilization through an autophagic and endocytic pathway (Politi et al. 2014). Although such mechanisms promote mitochondrial genetic homogeneity, paternal leakage has been documented in many animal species, both in natural populations and in laboratory intraspecific and interspecific crosses (for a broad review of species with paternal leakage see (Camus et al. 2022)). It seems that when carefully investigated, paternal leakage is very often detected. Most reports of paternal leakage provide only qualitative data for its presence or absence. Quantitative estimates are rare, but Wolff et al. (2013) quantified paternal leakage at 0.66% in *D. simulans* laboratory crosses (Wolff et al. 2013), and Nunes et al. (2013) estimated ∼6% in natural populations of *D. melanogaster* (Nunes et al. 2013). Although estimates vary and paternal leakage is frequently observed, the prevailing view is that paternal leakage is a primarily stochastic phenomenon.

Previous work demonstrated sex-specific paternal leakage (Dokianakis and Ladoukakis 2014) and family-level variation in heteroplasmy (Polovina et al. 2020) in crosses involving laboratory *Drosophila* strains. These observations suggest that paternal mtDNA leakage may not be a purely stochastic phenomenon. Here, we test the hypothesis that paternal mtDNA leakage is a nonrandom trait with a heritable genetic component associated with maternal lineages. To address this question we examined paternal leakage in multiple *D. simulans* isofemale lines derived from natural populations and therefore retaining naturally occurring genetic variation rather than in long-term laboratory stocks. Because all crosses were performed using males from a single *D. mauritiana* strain, variation among crosses could be attributed primarily to differences among maternal lineages. This design allowed us to determine whether naturally derived maternal lineages of *D. simulans* consistently differ in their propensity for paternal mtDNA leakage and whether natural populations exhibit greater variation in leakage than laboratory strains. Our results indicate that paternal leakage is highly heritable and that natural isofemale lines exhibit greater variance in leakage than inbred laboratory strains. These findings provide evidence that paternal mtDNA leakage is genetically controlled and offer new insight into its evolutionary significance and potential role in mitochondrial genome maintenance.

## Material and methods

### Lines, families, and crosses

We collected *D. simulans* from Iraklio, Crete, and Greece in 2019. We established 30 isofemale lines by placing a single fertilized wild-caught female per vial and allowing its offspring to interbreed. After the lines had expanded sufficiently, they were treated with tetracycline for 2 generations to eliminate *Wolbachia* according to a standard protocol (Brennan et al. 2012). Following treatment, the lines were confirmed to be *Wolbachia*-negative using PCR with primers wolF (5′-TTGTAGCCTGCTATGGTATAACT-3′) and wolR (5′-GAATAGGTATGATTTTCATGT-3′). The lines were subsequently maintained for many generations (at least 3 years) without tetracycline treatment prior to the experiments reported here.

To study heteroplasmy we performed a set of interspecific crosses between females from 15 isofemale lines of *D. simulans* randomly selected from the 30 initial lines established from the wild population and a laboratory strain of *D. mauritiana* (strain 14021-0241.151; Drosophila Species Stock Center), thereby keeping the paternal genetic background constant across crosses. The *D. simulans* isofemale lines were *siII* mitotype and the *D. mauritiana* strain was *maII* mitotype.

We performed 2 types of crosses. First, for the family crosses we crossed each female from the *D. simulans* lines with 3 *D. mauritiana* males. For each isofemale line we attempted to establish 10 independent family crosses (150 families in total), of which 118 produced F1 progeny and were included in the analysis. Families were treated as independent replicate crosses, nested within isofemale lines in subsequent statistical analyses. Second, for the line-specific crosses we crossed 10 females per *D. simulans* isofemale line with 30 *D. mauritiana* males. The males originated from the same laboratory strain but were different individuals across crosses. We have used the ratio 3 males to 1 female because it is common in interspecific crosses (eg Hammerle and Ferrus (2003), Sherengul et al. (2006)  Gerard and Presgraves (2012)). After the collection of virgin females we kept them in 25 °C for 3 to 5 d, to make sure that they were truly virgin. The age of the males varied as we did not collect newly born males but they were less than 10 d old. We also kept males for 3 to 5 d in 25 °C to confirm the absence of females. The crosses were performed in 25 °C with 12-h light period. Crosses were kept in the same vials until larvae appeared (approximately 3 to 4 d), after which they were transferred to fresh vials. This transfer was repeated 3 times. F1 hybrids were collected approximately 14 d after establishment of the cross or after transfer to fresh vials. Thus, individuals were collected at comparable developmental stages across families and lines. The F1 hybrids were stored in absolute ethanol (a schematic overview of the experimental design is shown in Fig. 1). Reciprocal crosses between *D. mauritiana* females and *D. simulans* males were previously shown not to produce detectable paternal mtDNA leakage in this species combination (Dokianakis and Ladoukakis 2014), and therefore the present design focused on variation among maternal *D. simulans* lines.

**Fig. 1.**
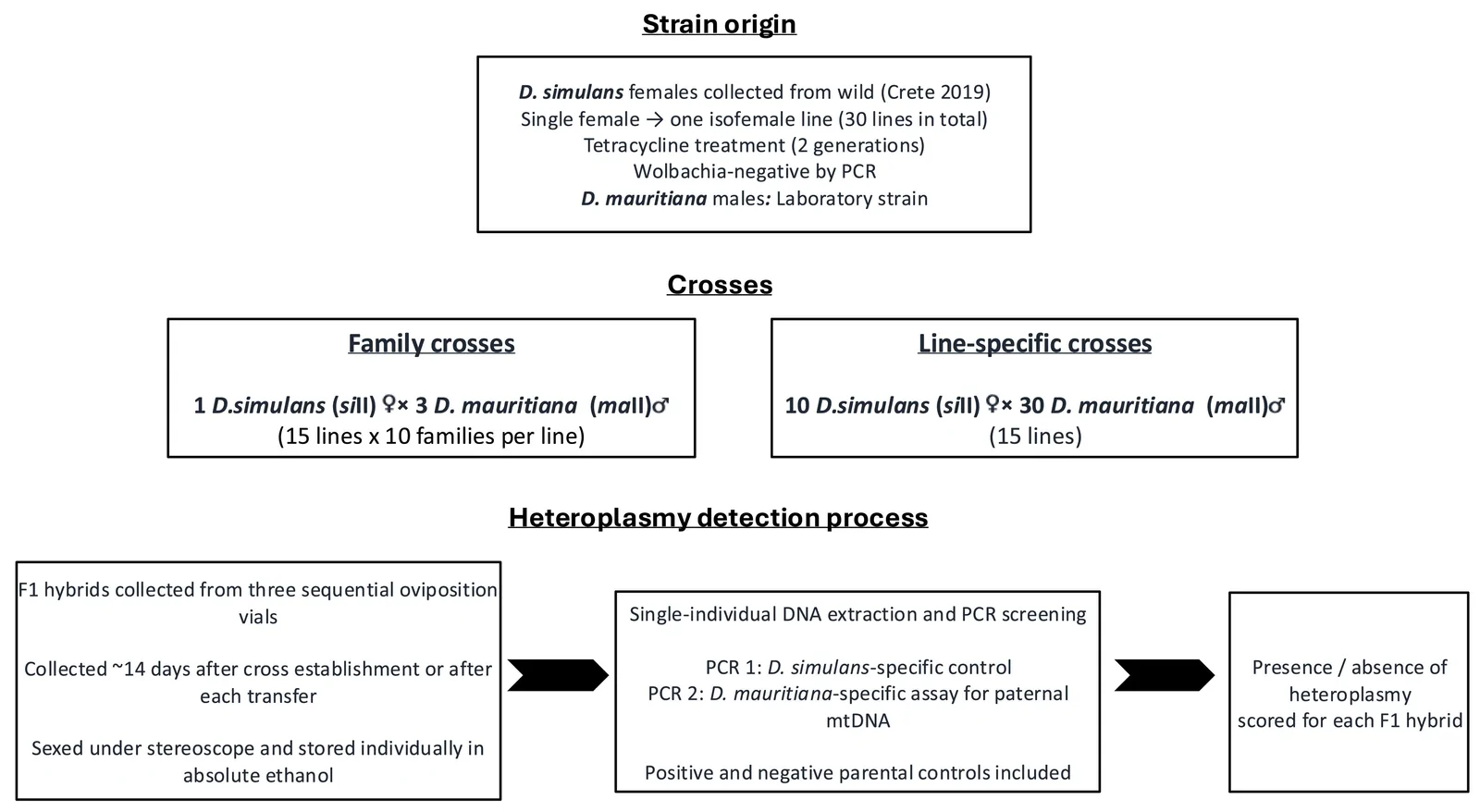
Experimental design for detecting paternal leakage.

### Heteroplasmy detection and statistical analysis

Total DNA was extracted from single individuals using the protocol described in (O'Neill et al. 1992). The sex of each individual was determined under a stereoscope prior to DNA extraction.

To detect heteroplasmy, 2 PCR reactions were performed for each individual. The first one was *D. simulans* specific and was used to confirm that the quality of the DNA of the individual was good enough to give PCR product. Individuals that produced no product with this PCR were excluded from further analysis. The primers used for this reaction were:

F: 5′-CCC TGA TAT AGC ATT CCC G-3′

R: 5′-GTT AAC CCC CCT ACT GTA-3′

Annealing temperature was 56 °C.

The second PCR reaction was *D. mauritiana* specific and was used to detect paternal mtDNA in the hybrids. The primers for this reaction were:

F: 5′-CCC AAC AAA TGA ATT AGC AAC-3′

R: 5′-TTA GAT TTA GGT GAA TTT GGT ATA-3′.

The annealing temperature was 50 °C. PCR products were run in 1% w/v agarose gel and were visualized with ethidium bromide staining.

For each PCR we used positive and negative controls from the strains that we have used as parental, eg, when we applied the first PCR (*D. simulans*) we used as positive control the maternal strain *D. simulans* and as negative control the paternal strain *D. mauritiana*. Detection of heteroplasmy was based on species-specific PCR amplification of *D. mauritiana* mtDNA and therefore reflects the presence or absence of paternal mtDNA rather than differences in total mtDNA copy number among individuals or maternal isofemale lines. For graphical presentation of heteroplasmy frequencies, uncertainty around proportions was estimated using Wilson binomial 95% confidence intervals.

To investigate the effects of sex, family, and line on the variation of the presence/absence of heteroplasmy, we fitted a generalized linear mixed model (GLMM) with a binomial distribution and a logit link function using the glmer function in the lme4 package in R (Bates et al. 2015). To test for potential interactions we fitted 3 models to include interactions with any of the 3 parameters (sex, family, and line). Because only the sex × family interaction was significant, the final model contained as random effects line, family, and sex × family.

We compared the full model (with the sex × family interaction) with reduced models using likelihood ratio tests (LRTs). LRTs were used to assess the significance of each random effect and interaction term.

### Variance partitioning

To quantify the proportion of variance explained by each factor, we extracted the variance components from the final model. The total variance was calculated as the sum of the variances of the random effects and the residual variance (assumed to be π^2^/3 for binomial models with a logit link). The proportion of variance explained by each factor was computed as:

Proportion of variance = *σ*^2^_Factor_/*σ*^2^_Total_.

## Results

### Effect of sex, line, and family on the proportion of heteroplasmic hybrids

To investigate whether heteroplasmy caused by paternal leakage in Drosophila hybrids has a genetic basis, we conducted a nested experiment using 15 isofemale lines of *D. simulans* (mitotype *siII*) collected from a natural population from Crete, Greece, encompassing 118 families derived from these lines. We assessed heteroplasmy by detecting paternal mtDNA in F1 offspring produced from crosses between *D. simulans* females and *D. mauritiana* males.

We analyzed a total of 2,443 F1 hybrid individuals (1,334 females and 1,109 males). Among them, 17 females (1.27%) and 553 males (49.86%) exhibited heteroplasmy, yielding an overall heteroplasmy frequency of 23.3% across sexes (Table 1). While paternal leakage was rare in females, it was highly prevalent in males (Fig. 2) as it has been observed in previous studies (Dokianakis and Ladoukakis 2014; Polovina et al. 2020).

**Fig. 2.**
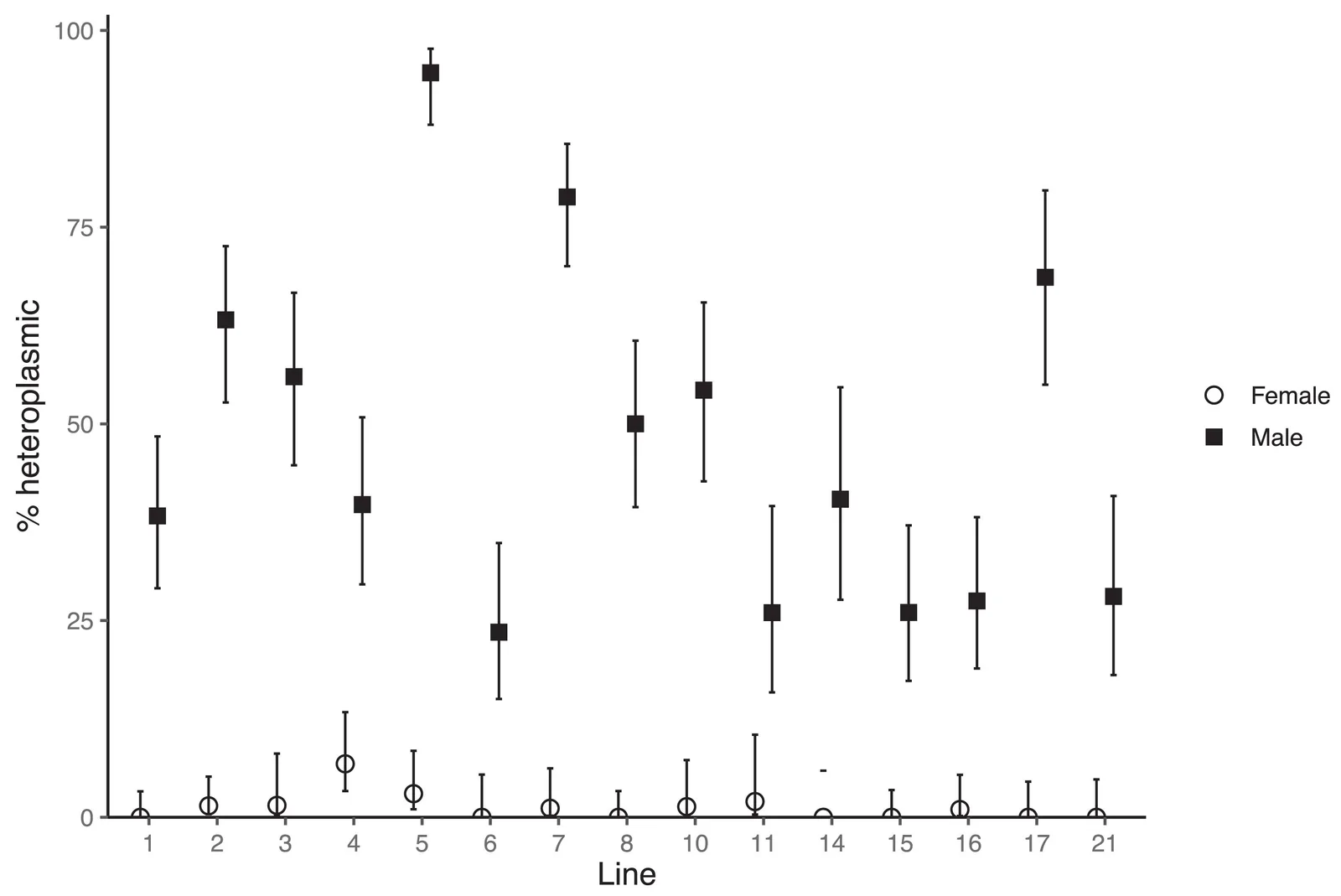
Heteroplasmy frequencies in male and female F1 hybrids across *D. simulans* isofemale lines. Points represent the observed proportion of heteroplasmic individuals for each sex within each line. Open circles indicate females and filled squares indicate males. Error bars represent Wilson 95% binomial confidence intervals.

**Table 1.** Heteroplasmy counts and percentages by line and sex.

		Females			Males			All		
line	No of families	Heter.	Total	%	Heter	Total	%	Heter.	Total	%
**5**	**10**	**3**	**100**	**3.00**	**88**	**93**	**94.62**	**91**	**193**	**47**.**15**
**7**	**10**	**1**	**87**	**1**.**15**	**82**	**104**	**78**.**85**	**83**	**191**	**43**.**46**
**3**	**7**	**1**	**66**	**1**.**52**	**42**	**75**	**56**.**00**	**43**	**141**	**30**.**50**
**10**	**6**	**1**	**74**	**1**.**35**	**38**	**70**	**54**.**29**	**39**	**144**	**27**.**08**
17	7	0	81	0.00	35	51	68.63	35	132	26.52
2	8	2	137	1.46	55	87	63.22	57	224	25.45
8	9	0	111	0.00	41	82	50.00	41	193	21.24
4	8	7	103	6.80	31	78	39.74	38	181	20.99
14	6	0	61	0.00	19	47	40.43	19	108	17.59
1	9	0	113	0.00	36	94	38.30	36	207	17.39
**11**	**5**	**1**	**50**	**2**.**00**	**13**	**50**	**26**.**00**	**14**	**100**	**14**.**00**
**16**	**9**	**1**	**101**	**0**.**99**	**22**	**80**	**27**.**50**	**23**	**181**	**12**.**71**
**21**	**8**	**0**	**76**	**0**.**00**	**16**	**57**	**28**.**07**	**16**	**133**	**12**.**03**
**6**	**8**	**0**	**67**	**0**.**00**	**16**	**68**	**23**.**53**	**16**	**135**	**11**.**85**
**15**	**8**	**0**	**107**	**0**.**00**	**19**	**73**	**26**.**03**	**19**	**180**	**10**.**56**
**Total**	**118**	**17**	**1334**	**1**.**27**	**553**	**1109**	**49**.**86**	**570**	**2443**	**23**.**33**

Entries are number heteroplasmic (Heter.), number assayed (total), and percent heteroplasmic (%). Values are shown for females, males, and all individuals. Lines in bold were used in the follow-up experiment (see Table 2).

We established families by mating single *D. simulans* females to up to 3 *D. mauritiana* males (Supplementary Table 1). In total, we produced 118 families across lines (median = 8 families per line). We assessed heteroplasmy in 4 to 26 females (median = 10) and 2 to 23 males (median = 10) per family. Heteroplasmy frequency varied widely across families, ranging from 0% to 54% in females (median = 0%), 0% to 100% in males (median = 50%), and 0% to 65% when sexes were combined (median = 23%).

At the line level, pooling all families per isofemale line revealed substantial variation in heteroplasmy. Across lines, heteroplasmy ranged from 0% to 6.7% in females (median = 0.9%), 23% to 95% in males (median = 40%), and 10% to 47% when sexes were combined (median = 21%) (Table 1).

We applied a GLMM to assess sources of variation. Sex had a highly significant effect (*P* < 2 × 10^−16^), with males being ∼654 times more likely to exhibit heteroplasmy than females. Both line and family contributed significantly to variation (line: *P* = 7.25 × 10^−6^; family: *P* < 2.2 × 10^−16^). Additionally, a strong sex × family interaction (*P* = 4.29 × 10^−10^) was detected, showing that the impact of sex differed among families. In contrast, line × sex and line × family interactions were not significant.

Variance partitioning revealed that “line” explained 15.4% of the total variance, “family” accounted for 5.5%, and the “sex × family” interaction explained 38.6%. The remaining 40.4% of variance remained unexplained. Because all crosses used males from a single laboratory strain and females of controlled age, this family-level variation most likely reflects structured maternal genetic effects rather than uncontrolled paternal or environmental differences among crosses. These findings support a strong genetic component at the line level, with lines explaining a greater proportion of variance than families.

### Heritability of heteroplasmy

We estimated the heritability of paternal leakage comparing variance across families within isofemale lines and not across isofemale lines themselves, which may be problematic (David et al. 2005). We estimated broad-sense heritability using 2 complementary approaches. We used a GLMM with “family” as a random effect which yielded an estimate of H^2^ = 0.464 ± 0.034 (95% confidence interval (CI): 0.398 to 0.533), representing the variance explained solely by family differences. We also used a variance decomposition approach, which defined genetic variance as the sum of the “family” and “family × sex” components, and produced a higher estimate of H^2^ = 0.742 ± 0.035 (95% CI: 0.673 to 0.807). This difference reflects that the variance decomposition approach incorporates additional interaction effects as part of genetic variance, while the GLMM approach attributes genetic variance only to “family” effects. Both approaches indicate that paternal leakage and heteroplasmy are strongly heritable traits.

### Replication of the experiment

To validate our findings, we performed a second experiment focusing on the 4 lines with the highest heteroplasmy (lines #5, 7, 3, 10-“high heteroplasmy”) and 4 with the lowest heteroplasmy (lines #15, 6, 21, 16; line #6 replaced by line #11 due to technical issues-“low heteroplasmy”) (Table 2). In the first experiment, heteroplasmy in the high heteroplasmy group ranged from 27.1% to 47.2%, while in the low group ranged from 10.6% to 14%. In the follow-up experiment, we mated approximately 10 females per *D. simulans* isofemale line with 30 males from *D. mauritiana* (ratio: 1 female to 3 males). We analyzed 20 to 62 females (total = 314 individuals) and 10 to 59 males (total = 272 individuals) across these 8 lines. The results were consistent with the first experiment because the high-heteroplasmy group retained significantly elevated heteroplasmy levels (33% to 51%) compared to the low heteroplasmy group (7% to 28%) (Table 2, Fig. 3).

**Fig. 3.**
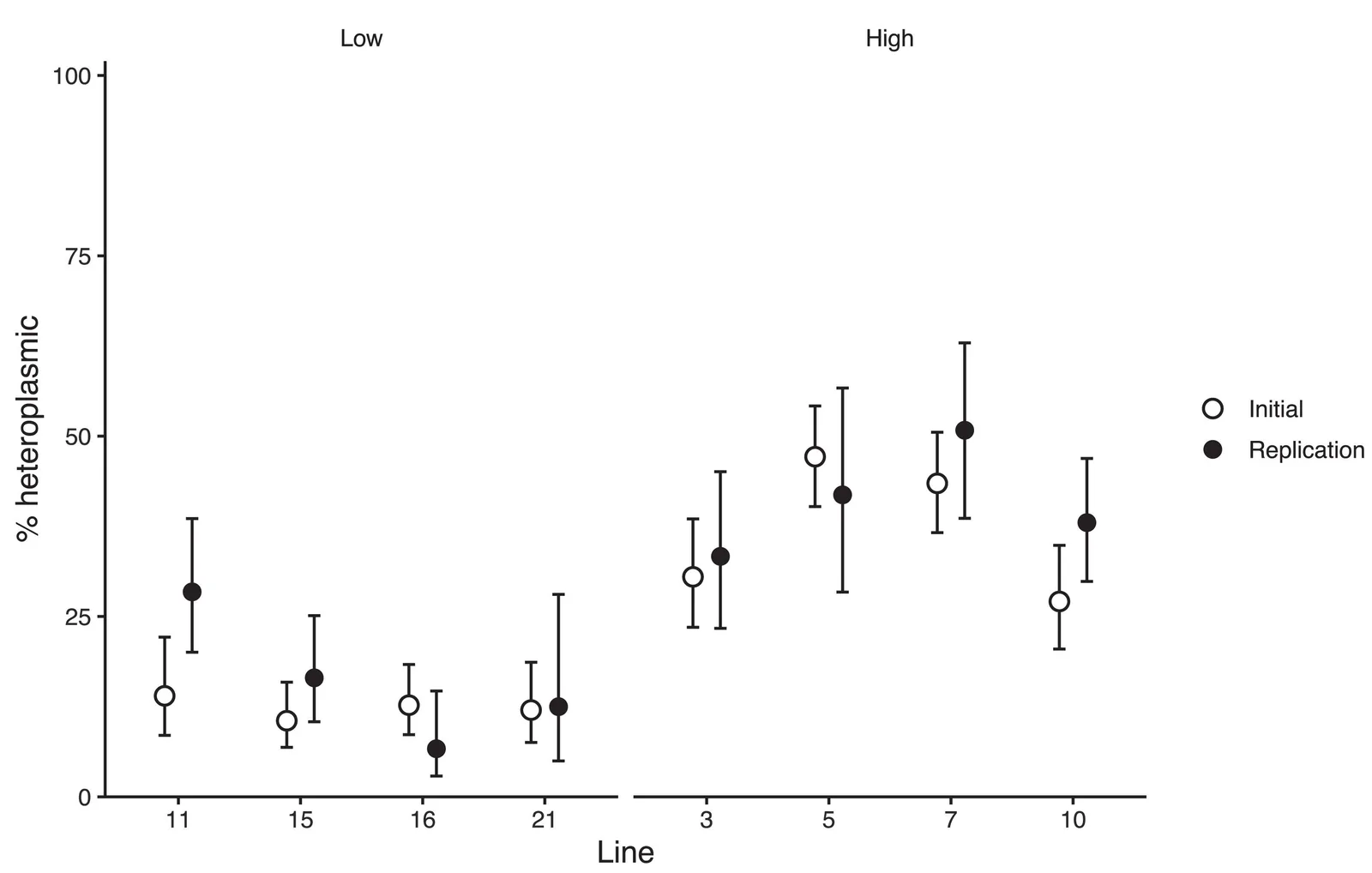
The overall level of heteroplasmy in “low” and in “high” isofemale lines in the initial and in the replication experiment. Points represent the observed proportion of heteroplasmic individuals. Error bars indicate Wilson 95% binomial confidence intervals.

**Table 2.** Replicate experiment of heteroplasmy in lines with extreme phenotypes.

	Female			Male			All		
line	Heter.	Total	%	Heter	Total	%	Heter.	Total	%
7	0	27	0.00	31	34	91.18	31	61	50.82
5	0	20	0.00	18	23	78.26	18	43	41.86
10	1	62	1.61	45	59	76.27	46	121	38.02
3	1	36	2.78	22	33	66.67	23	69	33.33
11	0	48	0.00	25	40	62.50	25	88	28.41
15	1	53	1.89	15	44	34.09	16	97	16.49
21	0	22	0.00	4	10	40.00	4	32	12.50
16	0	46	0.00	5	29	17.24	5	75	6.67
**Total**	**3**	**314**	**0**.**96**	**165**	**272**	**60**.**66**	**168**	**586**	**28**.**67**

Isofemale lines identified in Table 1 as high- (top block: lines 7, 5, 10, 3) or low-leakage (bottom block: lines 11, 15, 21, 16) were re-assayed in an independent experiment. For each line and sex (Female, Male) and for pooled sex (All) the number heteroplasmic (Heter.), the number examined (Total), and the percent heteroplasmic (%) are reported.

Ranking shifts existed within the 2 groups. For instance, although lines #5 and #7 consistently exhibited the highest heteroplasmy rates in both experiments, in the first experiment line #5 had higher heteroplasmy levels (47.15%) than line #7 (43.46%) whereas in the second experiment the heteroplasmy levels for these lines were 41.86 and 50.82, respectively. Also, line #15 exhibited the lowest heteroplasmy level in the first experiment (10.56%) whereas in the second experiment line #16 showed the lowest heteroplasmy level (6.67%). Despite the ranking shifts the overall grouping of the 8 lines to high and low heteroplasmy groups persisted. This reproducibility strongly supports a genetic basis for heteroplasmy.

### Comparison between natural and laboratory populations

We compared the results of this study with a previous experiment (Polovina et al. 2020) that examined heteroplasmy using females derived from a laboratory population rather than natural isofemale lines. Notably, the *D. mauritiana ma*II strain (14021-0241.151) used as the paternal parent in the present study was also one of the paternal strains used in Polovina et al. (2020), facilitating comparison between the 2 studies. Given that natural populations typically harbor greater genetic diversity, we hypothesized that if paternal leakage (and the subsequent heteroplasmy) had a genetic basis the diversity would be greater in natural populations than in laboratory populations. To test this, we computed the coefficient of variation (CV) for heteroplasmy presence/absence across families in both experiments. The CV was significantly higher in families from natural populations compared to those from laboratory populations for both males and females (Fligner–Killeen test, males: *P* = 1.734 × 10^−8^, females: *P* = 0.01948).

This result indicates that paternal leakage behaves as a quantitative genetic trait, having greater variability in the genetically diverse natural populations than in the inbred laboratory strains.

## Discussion

### Genetic component of paternal mtDNA leakage among maternal lineages

By measuring paternal mtDNA leakage in hybrids from crosses between *D. simulans* and *D. mauritiana*, we showed that the presence or absence of leakage has a strong genetic component across isofemale lines and families. In fact, using a single paternal strain ensured that the paternal genetic background remained constant across crosses, allowing differences in leakage levels to be attributed to variation among maternal isofemale lines. The same isofemale lines repeatedly exhibited consistently low or high leakage. Also, the heritability of paternal leakage across families exceeded 0.46. This heritability estimate is higher than those reported for traits such as body size and body shape (Yassin et al. 2022). Moreover, variation in paternal leakage was greater among lines derived from natural populations than among inbred laboratory lines. This pattern is expected for traits whose phenotypes are at least partly genetically determined (Simoes et al. 2008; Houot et al. 2010; Turner et al. 2011; Gloria-Soria et al. 2019).

### Evidence that paternal mtDNA leakage is nonrandom

The genetic component detected across maternal lineages challenges the view that paternal leakage is a stochastic by-product of imperfect mechanisms enforcing strict maternal inheritance. That current view is largely based on experimental observations of heteroplasmy transmission rather than on measurements of paternal leakage per se. Most studies of heteroplasmy across generations in mice, humans, and Drosophila (Gyllensten et al. 1991; Jenuth et al. 1996; Wonnapinij et al. 2008) focus on its transmission through mothers that are already heteroplasmic, rather than the heteroplasmy arising from paternal leakage. The dynamics of heteroplasmy transmitted by heteroplasmic females are strongly influenced by the mitochondrial bottleneck which takes place during oogenesis (Chinnery et al. 2000) and are therefore largely stochastic. Studies that directly measured paternal leakage indicate that it is nonrandomly distributed among individuals. In single-pair crosses between distinct mitotypes of *D. simulans*, Wolff et al. observed pair-specific paternal leakage (Wolff et al. 2013). Crosses in the angiosperm *Silene vulgaris* revealed significant differences in leakage rates among populations (Bentley et al. 2010). In addition, in family crosses between *D. simulans* and *D. mauritiana*, Polovina et al. (2020) documented heteroplasmy distributions attributable to paternal leakage that varied among families (Polovina et al. 2020). Finally, consistently higher frequency of paternal mtDNA leakage observed in males than in females was also reported in previous interspecific crosses (Dokianakis and Ladoukakis 2014). For this observation, one possible explanation is that mechanisms enforcing maternal inheritance of mtDNA may act more efficiently in females because females transmit mitochondria to the next generation, whereas males do not. Under this scenario, selection would favor stricter elimination of paternal mitochondria in females than in males. Consistent with this interpretation, paternal leakage in the present study showed not only lower frequency but also substantially lower variance among female hybrids than among males, suggesting tighter developmental or genetic control of paternal mitochondrial persistence in females. As a result, paternal mitochondria that escape elimination during early development may persist more frequently in males than in females, where leakage would have direct consequences for mitochondrial transmission to subsequent generations. Our results not only corroborate these indications of nonrandom paternal leakage but, through a systematic design, demonstrate that paternal leakage has a strong genetic component.

### Evolutionary implications of genetically controlled paternal leakage

Unless paternal leakage is merely a side effect of an unknown process or is pleiotropically linked to another function, its genetic control suggests that leakage itself may be functional. What might that function be? In mice, the coexistence of 2 wild-type mitotypes in a heteroplasmic state within an individual causes severe health disorders (Sharpley et al. 2012; Lechuga-Vieco et al. 2022). Such deleterious interactions between wild-type mtDNA haplotypes support the evolution of uniparental inheritance, but they do not explain why paternal leakage would be genetically regulated. A more plausible hypothesis is that leakage relates to the long-term maintenance of mtDNA. Under strict uniparental transmission, which restricts recombination, and given the elevated mutation rates observed in animals, mtDNA would be expected to succumb to the irreversible accumulation of deleterious mutations, a process known as Muller's ratchet (Muller 1964; Felsenstein 1974). Yet, mtDNA has remained functional since the origin of eukaryotes. This has been called mtDNA paradox (Loewe 2006). Theoretical analyses proposed that paternal leakage provides a solution because rare leakage does not undermine maternal transmission but allows occasional interlineage recombination, which can slow or halt Muller's ratchet (Hoekstra 2000; Loewe 2006). Indeed, very low levels of recombination are sufficient to counteract the ratchet (Gordo and Charlesworth 2000).

If this is the role of paternal leakage, it would mirror the role of mutation in genomes. The rate of paternal leakage appears to be genetically controlled like mutation rate (Lynch 2008; Lynch et al. 2016). This is consistent with the fundamental role of mutation in evolution which should be balanced because too many mutations would threaten the fidelity of genetic transmission across generations, whereas too few would reduce variation and hence adaptability. By analogy, tightly regulated paternal mtDNA leakage could permit enough recombination to sustain mitochondrial genome integrity without undermining the benefits of its maternal inheritance.

### A potential mechanism controlling paternal mtDNA leakage

In *Drosophila melanogaster*, paternal mtDNA is normally eliminated in 2 sequential steps: first during spermatogenesis, when mtDNA is removed from developing spermatids and second after fertilization through destruction of paternal mitochondria in the embryo (DeLuca and O'Farrell 2012; Politi et al. 2014; Wang et al. 2025). Paternal leakage therefore likely reflects incomplete elimination at one or both of these stages. Because hybridization can disrupt the efficiency of these elimination processes, our experiments were performed in hybrids, which is a situation uncommon in nature. Yet, the genetic component of paternal leakage was strongly evident. This implies that genetic control of leakage also operates in natural populations. It has been proposed that leakage is more frequent in hybrids than in intraspecific crosses because paternal mitochondria are less efficiently recognized in fertilized eggs (Rokas et al. 2003). If leakage were simply a by-product of hybridization, it should be entirely random, yet it is not. We therefore propose a 2-step control mechanism. First, sperm mtDNA is recognized and is eliminated shortly after fertilization or is eliminated from the sperm during spermatogenesis depending on the mechanism that each species uses to ensure maternal transmission. Some mitochondria could escape this step stochastically. Second, an independent mechanism governs the proliferation and/or persistence of any paternal mitochondria that survive the initial elimination. Variation in paternal leakage among maternal lineages is therefore more consistent with differences affecting this second step than with differences in the initial stochastic escape from elimination. In hybrids, recognition at step 1 is compromised because the signal in sperm and the recognition machinery in the egg come from divergent genetic backgrounds, allowing more paternal mitochondria to persist (Rokas et al. 2003). Step 2 then regulates their propagation or survival. Consequently, nonhybrids should display fewer detectable paternal mitochondria than hybrids simply because fewer organelles pass the first filter. A 2-step mechanism has been proposed for Drosophila (DeLuca and O'Farrell 2012; Politi et al. 2014; Wang et al. 2025) and for the Japanese rice fish *Oryzias latipes* (Nishimura et al. 2006). Whether the level of paternal leakage is related to the variety of mechanisms that ensure maternal inheritance of mtDNA remains to be tested experimentally. An important next step will be to identify the genetic factors underlying the variation in paternal leakage documented here. Because the *D. simulans*–*D. mauritiana* system consistently exhibits detectable paternal leakage, genetic approaches based on this system may provide a suitable framework for identifying the loci involved.

### Evolutionary implications of genetic control of paternal mtDNA leakage

The present study demonstrates that paternal mtDNA leakage is genetically controlled but does not identify the selective forces responsible for maintaining such variation. The evolutionary scenarios discussed above should therefore be viewed as hypotheses rather than conclusions directly supported by our data. If paternal leakage were consistently advantageous to the organism, it might be expected to become more common in natural populations. Conversely, if it were consistently detrimental, purifying selection might be expected to eliminate it. Instead, its low but persistent frequency across many taxa is consistent with the idea that leakage represents a compromise between preserving strict maternal inheritance, thereby avoiding mitochondrial conflict, and allowing occasional mitochondrial recombination that may contribute to the long-term maintenance of mitochondrial genomes. Under this interpretation, the evolutionary consequences of leakage may not be adequately captured by conventional measures of organismal fitness, such as viability, lifespan, or fecundity. Moreover, comparisons between naturally occurring high- and low-leakage lines would be difficult to interpret because these lines differ genetically at many loci. Consequently, any observed fitness differences could reflect the underlying genetic backgrounds rather than leakage itself.

## Supplementary Material

jkag199_Supplementary_Data

## Data Availability

All data supporting the results of this study are included in the manuscript and in Supplementary Table 1. Supplementary Table 1 contains family-level heteroplasmy counts and percentages used in the analyses. Supplemental material available at G3 online.
